# *Flos Lonicera* Combined with Metformin Ameliorates Hepatosteatosis and Glucose Intolerance in Association with Gut Microbiota Modulation

**DOI:** 10.3389/fmicb.2017.02271

**Published:** 2017-11-17

**Authors:** Na R. Shin, Shambhunath Bose, Jing-Hua Wang, AbuZar Ansari, Soo-Kyoung Lim, Young-won Chin, Han-seok Choi, Hojun Kim

**Affiliations:** ^1^Department of Rehabilitation Medicine of Korean Medicine, Dongguk University, Goyang, South Korea; ^2^NosQuest, Bundang-gu, South Korea; ^3^College of Pharmacy, Dongguk University, Goyang, South Korea; ^4^Department of Endocrinology, Dongguk University, Goyang, South Korea

**Keywords:** *Flos Lonicera*, metformin, metabolic syndrome, gut microbiota, hepatosteatosis

## Abstract

The gut microbiota is important in energy contribution, metabolism and immune modulation, and compositional disruption of the gut microbiota population is closely associated with chronic metabolic diseases like type 2 diabetes (T2D) and non-alcoholic fatty liver disease (NAFLD). Metformin (MET) and *Flos Lonicera* (FL) are common treatments for metabolic diseases in Western and Oriental medicinal fields. We evaluated the effect of treatment with FL and MET in combination on hepatosteatosis, glucose tolerance, and gut microbial composition. FL and MET were administered to Otsuka Long-Evans Tokushima Fatty (OLETF) rats, an animal model of genetic T2D and NAFLD. The FL+MET treatment reduced liver weight, serum cholesterol, insulin resistance, and hepatic MDA level and modulated the gut microbial composition. More specifically, the genera of *Prevotella* and *Lactobacillus* were negatively associated with the body and liver weights, hepatic TG and TC content, and serum insulin level. However, the relative abundance of these genera decreased in response to the FL+MET treatment. Interestingly, pathway prediction data revealed that the FL+MET treatment attenuated lipopolysaccharide-related pathways, in keeping with the decrease in serum and fecal endotoxin levels. FL and MET in combination exerts a synergistic effect on the improvement of hepatosteatosis and insulin sensitivity in OLETF rats, and modulates gut microbiota in association with the effect.

## Introduction

Metabolic diseases are of major concerns in public health policy as they are associated with a broad spectrum of disease and complications including, cardiovascular disease (CVD), type 2 diabetes (T2D), hyperlipidemia, and cancer (Zhang et al., 2013). Notably, nonalcoholic fatty liver disease (NAFLD), a condition associated with excessive lipid accumulation in the liver, is strongly linked to T2D (Abu-Shanab and Quigley, 2010).

Metformin (MET), a first line clinical medicine for T2D, acts primarily by reducing hepatic glucose production and increasing glucose uptake (Bosi, 2009). A randomized controlled clinical trial has shown that MET is useful in the treatment of NAFLD (Bugianesi et al., 2005). However, compared with other diabetic agents, MET needs a higher dosage and longer treatment period to exert beneficial effects. Additionally, MET treatment is associated with a number of side effects such as, adverse lipid metabolism (Bosi et al., 2009), vitamin B12 deficiency (Bell, 2010), and digestive disorders (Bouchoucha et al., 2011). Therefore, MET is often administered in combination with other drugs including herbal products such as, vildagliptin (Bosi et al., 2009), compound K (Yoon et al., 2007), and *Vernonia amygdalina* (Michael et al., 2010) to potentiate the treatment efficacy and reduce the dosage and associated toxicity. However, to the best of our knowledge, no study has been conducted so far to evaluate the impact of MET and FL in combination on NAFLD.

*Flos Lonicera* (FL), an herbal medicine containing several active compounds such as, iridoid glucoside, chlorogenic acid, and caffeic acid (Peng et al., 2000; Wang et al., 2014), is widely used in east Asia as traditional treatment for many diseases. This herb possesses a number of beneficial therapeutic properties including cytoprotective, antimicrobial, antibiotic, antioxidative, and anti-inflammatory activities (Sulaiman et al., 2008). FL also possesses anti-diabetic activities and improves renal complications in streptozotocin-induced diabetic rat model (Tzeng et al., 2014; Han et al., 2015). FL is hepatoprotective (Teng et al., 2010) and thus can ameliorate nonalcoholic steatohepatitis (NASH) in a high-fat diet (HFD)-induced NAFLD model (Tzeng et al., 2015).

Gut microbiota is vital for metabolism of nutrients and energy production, and maintenance of balance with the host's metabolism and immune modulation (Flint et al., 2012). The composition of gut microbiota is significantly associated with metabolic syndromes, T2D, and NAFLD (Turnbaugh et al., 2006; Qin et al., 2012; Schnabl and Brenner, 2014; Song et al., 2014; Wang et al., 2014). An imbalance in the ratio of gut microbiota contributes to the onset and development of obesity, which is driven by a number of factors including promotion of energy harvest from diet, activation of systemic inflammation, and increase of fat deposition (Bajzer and Seeley, 2006; Tsai and Coyle, 2009). The fermentation of undigested carbohydrates by gut microbiota primarily produces acetate, propionate, butyrate, and lactate, which are the members of short chain fatty acids (SCFAs) (Cani and Knauf, 2016). SCFAs modulate the host metabolism through several mechanisms (Hur and Lee, 2015). For example, the signaling of SCFAs through G protein-coupled receptor 41 (GPR41) on enteroendocrine cells induces secretion of peptide YY (PYY) that inhibits gut motility, augments intestinal transit rate, and decreases the harvest of energy from the diet. Gut microbiota also strongly suppresses the expression of fasting-induced adipose factor (Fiaf) in the ileum, which inhibits lipoprotein lipase (LPL) activity and prevents fat storage in the white adipose tissue. Furthermore, SCFAs-mediated induction of GPR43 impairs insulin signaling in the adipose tissue, and subsequently blocks fat accumulation. SCFAs also induce intestinal gluconeogenesis (IGN) through a gut-brain neural circuit, which can boost glucose metabolism and suppress food intake (Hur and Lee, 2015).

MET modulates the population of gut microbes such as, *Akkermansia* spp. and *Clostridium* spp. in a mouse model of HFD-induced obesity, which is associated with the improvement of metabolic parameters including glucose homeostasis (Shin et al., 2013; Lee and Ko, 2014). Both unfermented and fermented FL formulations could significantly improve HFD-induced obesity and related endotoxemia (Wang et al., 2014). More specifically, modulation in the distribution of gut microbiota, especially restoration of relative abundance of *Akkermansia* spp. and the *Bacteroidetes*/*Firmicutes* ratio by the above two formulations could be vital in combating HFD (Wang et al., 2014). However, no study assessed the effect of MET and FL treatments in combination and the potential synergistic beneficial impact on the gut microbial niche in disease states like metabolic disorders and liver disease. This prompted us to conduct the present study where with the help of predicted metabolic pathways (Moran and Bi, 2006; Linden et al., 2014), we evaluated whether there is a synergic effect of MET and FL in Otsuka Long-Evans Tokushima Fatty (OLETF) rats, an animal model of genetic obesity, T2D and NAFLD, and, if so, the intrinsic molecular mechanisms and the role of gut microbiota.

## Materials and methods

### Preparation of FL extract

Dried FL flowers in powdered form were procured from the medical supply store of Dongguk University International Hospital (Goyang, Republic of Korea). FL was extracted from powdered FL (1.4 kg) after mixing with ethanol (6 L) at 100°C for 3 h using a reflux condenser. The collected liquid extract was evaporated to dryness by rotary evaporator (EYELA N-1200A, EYELA, Tokyo, Japan), freeze-dried using a lyophilizer (Bondiro, IlshinBioBase, Dongducheon, Republic of Korea), and kept at −80°C until further use. The yield of FL was 8.7% (w/w).

### High performance liquid chromatography (HPLC)-based analysis of FL

Chromatographic analysis of FL was performed using an HPLC system (1260 infinity, Agilent Technologies, Santa Clara, CA, USA) equipped with a UV detector, an online degasser, and an autosampler. The mobile phase A was water:*o*-phosphoric acid (99.5:0.5, v/v) and mobile phase B was acetonitrile:water:*o*-phosphoric acid (50:49.5:0.5, v/v). The separation was carried out through an Eclipse XDB-C18 column (5 μm, 250 × 4.6 mm. Agilent Technologies, USA) at 25°C maintaining a flow rate of mobile phase at 1 ml/min with the following gradient: 0–5 min, 90% A, 5–18 min 90–18% A for 18 min, and 18–90% A for 0.1 min). The detection was performed at 327 nm using chlorogenic acid and caffeic acids (Sigma Aldrich, St. Louis, MO, USA) as standards.

### Analysis of total flavonoid content

Total flavonoid content was analyzed by aluminum chloride reaction methods. FL extract was reacted with 5% NaNO_2_ for 5 min and added to 10% AlCl_3_ for 5 min. The reaction was stopped by adding 1 M NaOH and an absorbance value was measured at 570 nm on a microplate reader (Spectramax Plus, Molecular Devices, Sunnyvale, CA, USA). The Catechin (Sigma Aldrich, USA) was used as standard.

### Cell culture condition

C2C12 cells were cultured in DMEM (Welgene, Daegu, Republic of Korea) supplemented with 1% FBS (HyClone, Logan, UT, USA) and 1U penicillin/streptomycin (Life Technologies, Carlsbad, CA, USA). INS-1 cells were cultured in RPMI 1640 (Gibco, Grand Island, NY, USA) with 10% FBS, 10 mM HEPES (Sigma Aldrich, USA), 1 mM sodium pyruvate (Life Technologies, USA), 50 μM 2-mercaptoethanol (Sigma Aldrich, USA), and 1U penicillin/streptomycin. The growing conditions of both cells were 37°C in a humidified atmosphere of 5% CO_2_ in air.

### Analysis of glucose uptake by C2C12 cells

After the confluence of C2C12 reached 90%, the medium was removed and replaced with the differentiation medium (DMEM supplemented with 20 mM glucose and 2% horse serum; Gibco, USA) followed by another change on day 2. The medium was changed on day 4 to DMEM supplemented with 2.5 mM glucose (Sigma Aldrich, USA). Two hours after this change, the cells were treated with 0.75 mM MET (Sigma Aldrich, USA) either alone or in combination with FL (50, 100, 200 μg/ml) for 15 h. Finally, the cells were treated with 0.3 mM 2-deoxy-2-[(7-nitro-2,1,3-benzoxadiazol-4-yl)amino]-D-glucose (2-NBDG, Life Technologies, USA) for 1.5 h. The cells were then washed twice with DPBS. The glucose uptake was measured by detecting the fluorescence intensity of 2-NBDG at excitation and emission wave lengths of 485 and 535 nm, respectively, using a microplate reader (Molecular Devices, USA).

### Analysis of insulin secretion by INS-1 cells

After a 24 h culture of the INS-1 cells, the medium was replaced by 20 mM high glucose RPMI 1640 medium. The cells were treated with FL at 50, 100, and 200 μg/ml concentrations in absence or presence of 0.75 mM MET for 2 days. Following this, the cells were washed with modified KPBB-HEPES buffer (134 mM NaCl, 4.8 mM KCl, 1 mM CaCl_2_, 1.2 mM MgSO_4_, 1.2 mM KH_2_PO_4_, 5 mM NaHCO_3_, 10 mM HEPES, and 1 mg/mL BSA; pH 7.4) and then incubated in the same buffer supplemented with 20 mM glucose for 1.5 h. The supernatant was collected and centrifuged at 12,000 g for 10 min at 4°C and used for determination of insulin with an ELISA assay kit (Mercodia Rat Insulin Kit, Mercodia, Sweden). Cells were lysed in a lysis buffer [1% Triton-X, 20 mM HEPES, pH 7.4, 100 mM KCl, 2 mM EDTA, 1.0 mM PMSF, with protease inhibitors (Roche)]. Total protein of the cell extract was determined using a BCA protein assay kit (Thermo Scientific, Rockford, IL, USA).

### Animals and experimental schedule

Six-weeks-old male LETO and OLETF rats were purchased from Otsuka Pharmaceutical Co. (Tokushima, Japan) and acclimated for 6 weeks under 12 h light/dark cycle at constant temperature (25°C) and humidity (50–60%) with free access to chow diet and water. After acclimatization, OLETF rats were randomly divided into three groups (seven animals/per group) and treated as per the following specifications. MET group: treated with MET (100 mg/kg/day), FL+MET group: treated with FL (200 mg/kg/day) in combination with MET (100 mg/kg/day), and OLETF group: treated with water. The rats in LETO groups (seven animals) were also treated with water. The treatments were given orally (Food and Drug Administration, 2005) using water as the vehicle. The treatments were carried out six times a week for a period for 12 weeks. After the termination of experimental duration, all animals were subjected to overnight (12 h) fasting. The animals were sacrificed under Zoletil (Tiletamine-zolazepam, Virbac, Carros, France)-Rompun (xylazine-hydrochloride, Bayer, Leverkusen, Germany) combination anesthesia (1:1, v/v). Blood samples were collected from the central aorta and rapidly transferred into a BD Vacutainer (BD, Franklin Lakes, NJ, USA). The liver was excised quickly, washed in ice-cold PBS, pH 7.4, dried, weighed and stored at −80°C until used. For future histological analyses, portions of the liver were fixed immediately in 10% formalin (Junsei, Tokyo, Japan; for paraffin sectioning) and FSC 22® frozen section media (Leica Biosystem, Richmond, IL, USA; for frozen sectioning) and stored at room temperature and −20°C, respectively for further analyses. Fresh stool samples were also collected and stored at −80°C. The blood samples were allowed to clot for 2 h at room temperature and then centrifuged at 3,000 g for 15 min. The sera were separated and stored at −80°C for further analyses.

The animal study was approved by the Institutional Animal Care and Use Committee (IACUC-2014-037) of Dongguk University and performed in accordance with the “Guide for the Care and Use of Laboratory Animals” (Institute of Laboratory Animal Resources, Commission on Life Sciences, National Research Council, USA; National Academy Press: Washington D.C., 1996).

### Oral glucose tolerance test (OGTT) and intraperitoneal insulin tolerance test (IPITT)

The animals were subjected to overnight (12 h) fasting and administered sterilized glucose solution (2 g/kg, Sigma Aldrich, USA) by oral gavage. The glucose levels of the blood samples collected from the tail vein were measured at five different time points (0, 0.5, 1, 1.5, and 2 h) using ACCU-CHEK Active (ACCU-CHEK, Mannheim, Germany). The IPITT was performed 2 days prior to the termination of animal experiment. The animals were subjected to overnight (12 h) fasting, then administrated with biosynthetic human insulin (0.75 U/kg, Eli Lilly and Company, Indianapolis, IN, USA; Um et al., 2004) by intraperitoneal injection. The glucose levels of the blood samples collected from the tail vein were measured at five different time points (0, 0.5, 1, 1.5, and 2 h) as described above for OGTT.

### Histology of liver

After embedding in paraffin blocks, formalin-fixed liver tissues were sectioned to 6 μm thickness on a microtome (Leica RM2235, Leica, Nussloch, Germany). The sections were placed on silicon coated glass slides (Leica Biosystem, Richmond, IL, USA), dried, deparaffinized with xylene and rehydrated in decreasing ethanol series. The sections were stained with 2.5% hematoxylin (Merck, Darmstadt, Germany) followed by counter-staining with 0.5% eosin (Sigma Aldrich, USA). The sections were dehydrated with an increasing ethanol series and xylene and mounted in a xylene-based mounting media (Histomount, Atlanta, GA, USA). Oil-Red-O staining was performed on liver tissues preserved in frozen section media as stated above. Freshly trimmed hydrated tissue sections were placed on silicon coated glass slides and fixed in 10% formalin for 5 min at room temperature. The sections were rinsed in running tap water for 10 min and immersed in 100% propylene glycon (Samchun, Gyeonggi-do, Republic of Korea) with two changes, then finally stained for 15 min with Oil-Red-O stain solution (Sigma Aldrich, USA; prepared in propylene glycon). The sections were rinsed in 85% propylene glycon for 3 min, rinsed in distilled water, stained in hematoxylin (Sigma Aldrich, USA), and eosin (Sigma Aldrich, USA) (H&E), and rinsed in distilled water for 5 min. The sections were mounted in aqueous glycerol (Samchun, Repubilc of Korea. Stained tissue sections were examined under an Olympus BX61 light microscope (Olympus, Tokyo, Japan) with 200X magnification. Images were captured (100 μm scale bar) using an Olympus DP70 digital camera (Olympus, Japan).

### Serum biochemical analyses

The serum triglyceride (TG), total cholesterol (TC), high-density lipoprotein cholesterol (HDL-C), glutamic oxaloacetic transaminase (GOT), and glutamic pyruvate transaminase (GPT) levels were determined using commercial enzymatic assay kits (Asan Pharmaceutical Co., Seoul, Republic of Korea) according to the kit manufacturer's instructions. The concentration of low-density lipoprotein cholesterol (LDL-C) was calculated using Friedewald formula, TC-HDL-(TG/5) (Friedewald et al., 1972). The serum insulin level was measured using a rat insulin ELISA kit (Mercodia, Uppsala, Sweden) as per kit manufacturer's protocol.

### Hepatic and fecal TG and TC content

Stored liver tissues (200 mg) were homogenized in 1 ml of distilled water. Stored fecal samples were freeze-dried using a lyophilizer (IlshinBioBase, Dongducheon, Republic of Korea) and dried fecal matter (100 mg) was homogenized in 1 ml distilled water. The homogenate was extracted with 5 mL chloroform-methanol (2:1) mixture and centrifuged at 7,000 g for 5 min. The chloroform layer was taken, dried, and resolved by isopropanol. Liver TG and TC levels were determined using commercial enzymatic assay kits (Asan Pharmaceutical Co., Seoul, Republic of Korea) as per instructions of kit manufacturer.

### Liver malondialdehyde (MDA) analysis

Liver tissues were homogenized using an Ultra-Turrax homogenizer (IKA, Staufen, Germany) in ice-cold 0.15 M KCl solution containing 1% phosphoric acid (Sigma Aldrich, USA). Thiobarbituric acid (Sigma Aldrich, USA) was added to the homogenate at a final concentration of 0.8% and the mixture was kept in a water bath at 90°C for 45 min. The mixture was placed on ice and centrifuged at 2,000 g for 15 min following which n-butanol (Sigma Aldrich, USA) was added. The supernatant was aspirated carefully and its absorbance was read at 535 nm on a microplate reader (Molecular Devices, USA). The hepatic MDA content was determined from a freshly prepared standard curve of 1,1,3,3,-tetraethoxypropane (Sigma Aldrich, USA) and normalized with the total tissue protein. Protein concentrations were determined using a BCA protein assay kit (Thermo Scientific, USA).

### Serum and fecal endotoxin analyses

Endotoxin levels in serum and fecal samples were determined using a Limulus Amebocyte Lysate (LAL) kit (ENDOSAFE, Charleston, SC, USA) according to the kit manufacture's protocol.

### Western blot

Stored liver tissues were homogenized in protein extraction solution (iNtRON Biotechnology, Inc., Gyeonggi-do, Republic of Korea) containing protease inhibitor (Sigma Aldrich, USA) and phosphatase inhibitor cocktail (GenDEPOT, Barker, TX, USA). Protein concentrations of the homogenates were determined using a BCA protein assay kit (Thermo Scientific, USA). Forty microgram of proteins was separated on a 10% SDS-PAGE gel provided with a constant voltage of 80 V, then transferred to a PVDF membrane (Amersham, Buckinghamshire, UK). The membranes were blocked with 5% skim milk (Becton Dickinson, Sparks, MD, USA) in Tris-buffered saline (Sigma Aldrich, USA). The membranes were incubated overnight with appropriate primary antibodies at 4°C. The immunoblots were washed three times in TBS buffer and then incubated with appropriate secondary antibodies for 1 h. The immunoblots were washed twice. The immunoblotted proteins were visualized using an ECL Western bolting luminal reagent, and the band intensities were quantified using a LAS-3000 Chemiluminescence detection system (Fujifilm, Tokyo, Japan). Primary antibodies were: anti-phosphorylated(p)-AMPK, anti-AMPK (Cell signaling, Danvers MA, USA), anti-SREBP-1c (Abcam, Cambridge, UK), anti-p-ACC (Cell signaling, USA), anti-HMGCoA reductase (Merck, Darmstadt, Germany), and anti-β-actin (Santa Cruz Biotechnology, Santa Cruz, CA, USA); secondary antibodies used were: HRP-conjugated mouse and rabbit IgGs (Santa Cruz Biotechnology, Santa Cruz, CA, USA).

### Sequencing

Genomic DNA from fecal samples was isolated using QIAamp stool DNA mini kit (QIAGEN, Hilden, Germany) following the kit manufacturer's instructions. The PCR of V1–V3 region of 16S rRNA gene sequences was performed using a C1000 Touch thermal cycler (Bio-Rad, Hercules, CA, USA). Amplicons were purified using a QIAquick PCR purification kit (QIAGEN, Germany). An equimolar concentration of each amplicon from different samples was pooled and purified using an Ampure bead kit (Agencourt Bioscience, Beverly, MA, USA) and quantified using a PicoGreen ds DNA assay kit (Invitrogen, Carlsbad, CA, USA). The mixed amplicons were amplified on sequencing beads by emulsion PCR. Sequencing reactions were performed using a Roche/454 GS Junior system (454 Life Sciences, Branford, CT, USA) according to the manufacturer's instructions. Raw sequence data are available in the European Nucleotide Archive (ENA) with accession numbers PRJEB19873.

### Sequence data analysis

Obtained sequence data were sorted by a unique barcode in the demultiplexing step, and low quality reads (average quality score <25 or read length <300 bp) were not considered for analysis. Sequences were assigned to the operational taxonomic units (OTUs; 97% identity; Greengenes database: http://greengenes.lbl.gov) followed by the selection of representative sequence using the Quantitative Insights into Microbial Ecology (QIIME) software package (Caporaso et al., 2010). Phylogenetic Investigation of Communities by Reconstruction of Unobserved States (PiCRUSt) was performed to identify functional genes in the sampled microbial community on the basis of the data in the Kyoto Encyclopedia of Genes and Genomes (KEGG) pathway database (Langille et al., 2013). To identify taxa with differentiating relative abundance in the groups, the LEfSe analyses were performed using the web-based program (http://huttenhower.sph.harvard.edu/galaxy; Segata et al., 2011) for which the following conditions were applied: the alpha value of the factorial Kruskal-Wallis test among classes was set to <0.05 and the threshold on the logarithmic LDA score for discriminative feature was set to >2.0.

### Statistical analysis

Data were expressed as means ± SD unless otherwise indicated. Statistical significance was calculated with one-way analysis of variance (ANOVA) followed by the Tukey *post-hoc* test. Significance was accepted at the level of *p* < 0.05, *p* < 0.01, or *p* < 0.001. Relationship strength between parameters was assessed using the two tailed Pearson's correlation test. The correlation was considered significant only when the absolute value of Pearson's correlation coefficient *r* was > 0.5.

## Results

### Chemical characterization of FL extract

HPLC analyses revealed that the acquisition rate of ethanol extract of FL was 13.41%, the chlorogenic acid content of FL was 29.8 μg/mg, and caffeic acid content was 2.3 μg/mg (Figure S1). The flavonoid content of FL analyzed by aluminum chloride reaction method was 109.3 μ g/mg.

### Combination of FL and MET treatment reduced the liver weight, improved hepatic histology, and suppressed hepatic lipid deposition in OLETF rats

**Figure 1:**
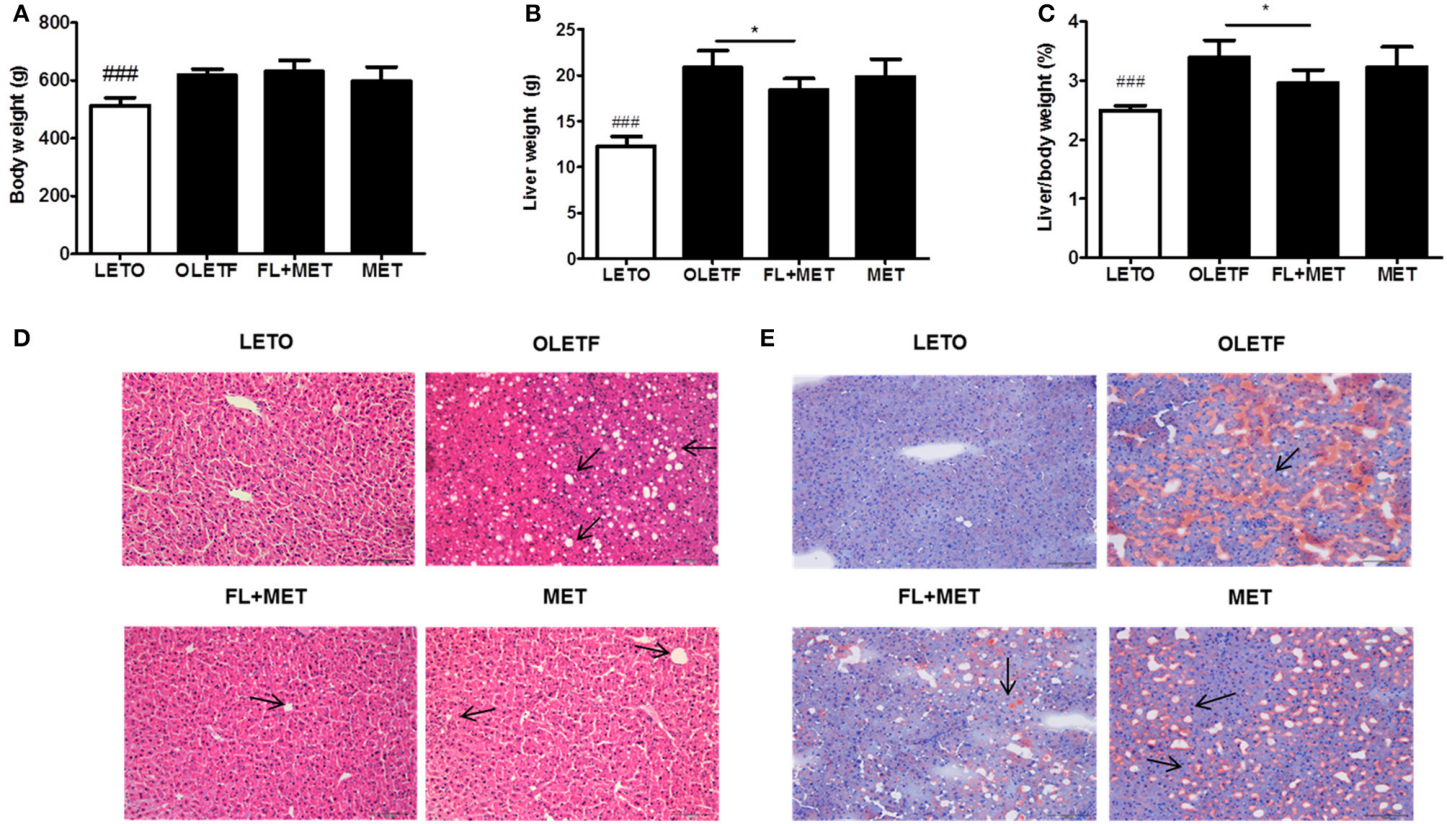
Effect of FL on the body and liver weights, histology, and fat accumulation in OLETF rats. The results depict body weight **(A)**; liver weight **(B)**; and relative liver weight **(C)**. Histopathological observations of the liver tissues stained with hematoxylin-eosin **(D)** and Oil Red O **(E)** were carried out under light microscopy with 200X magnification (scale bar 100 μm, arrows indicate the lipid droplets). Data are expressed as mean ± SD (*n* = 7). ^###^*P* < 0.001 vs. LETO group; ^*^P < 0.05 vs. OLETF group.

Although the body weight of OLETF rats did not change significantly in response to the treatment with FL and MET combination (FL+MET; Figure 1A), both the absolute and relative weights of liver of the animals in FL+MET group, but not in MET group, were significantly lower compared to OLETF rats (Figures 1B,C).

As expected, H&E histological staining revealed the normal structural integrity of liver in LETO group and hepatic steatosis (arrows) in OLETF group (Figure 1D). However, the extent of steatosis in the liver of FL+MET group was much lower compared to OLETF group. Oil-Red-O staining revealed the absence of lipid droplets in LETO group but presence of those in OLETF group (arrow, Figure 1E). However, the hepatic lipid deposition in OLETF rats was reduced remarkably in response to the treatment with both MET and FL+MET groups.

### Combination of FL and MET reduced serum and hepatic cholesterol and increased fecal cholesterol

**Figure 2:**
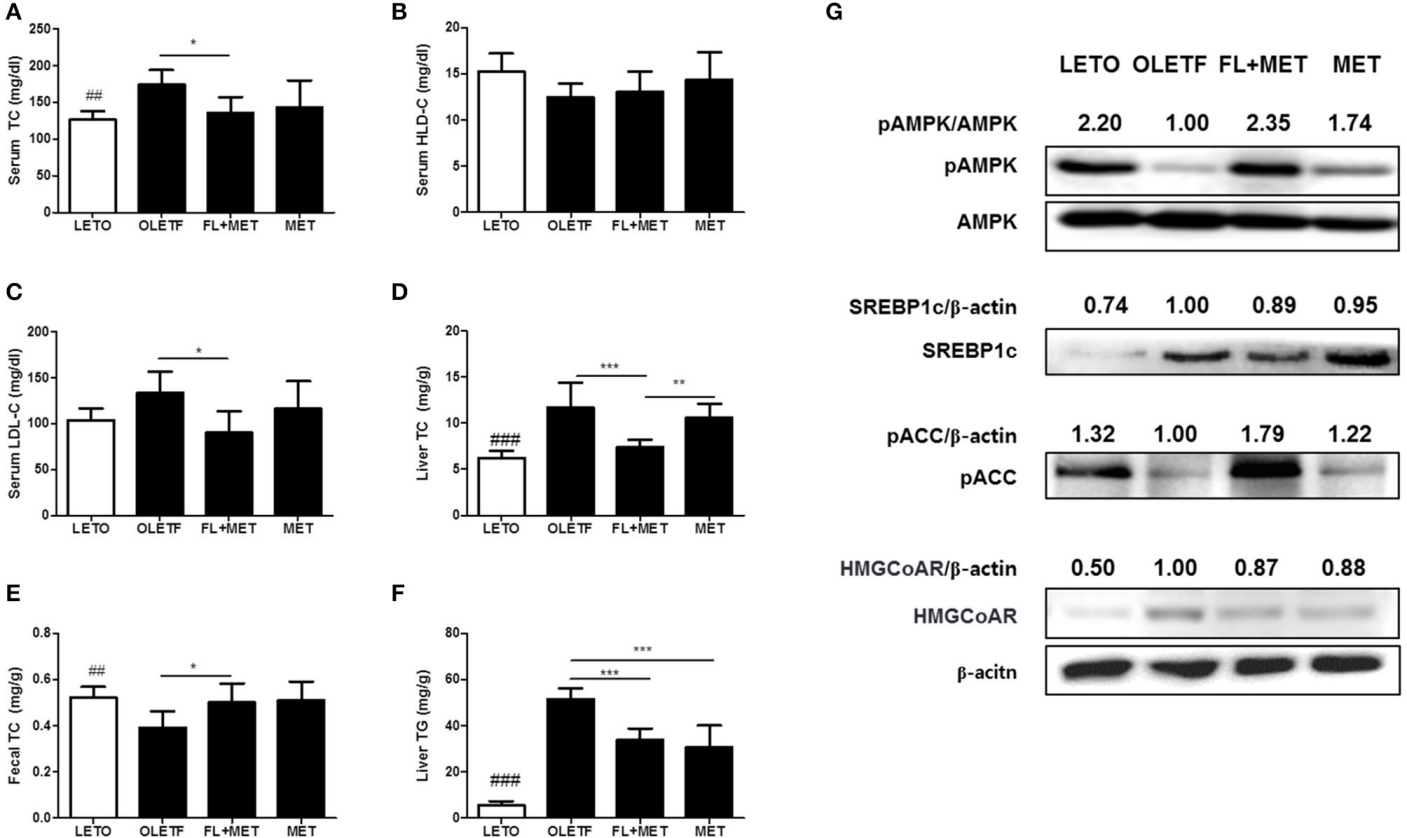
Effect of FL on lipid profiles and liver protein expression levels in OLETF rats. The concentration of total cholesterol **(A)**, HDL cholesterol **(B)**, and LDL cholesterol **(C)** in the serum of OLETF rats were measured. The content of and hepatic **(D)** and fecal total cholesterol **(E)** and hepatic TG **(F)** in OLETF rats were determined. The protein expression of SREBP-1c, HMGCoA reductase and phosphorylation levels of AMPK and ACC in the liver of OLETF rats were estimated by Western blotting. Representative blots from at least three individual experiments for each protein are shown **(G)**. The density of the bands in each blot was quantified by densitometric analysis and expressed as pAMPK/AMPK, SREBP-1c/β-actin, pACC/β-actin, and HMGCoA reductase/β-actin. Data are expressed as mean ± SD (*n* = 7). ^##^*P* < 0.01; ^###^*P* < 0.001 vs. LETO group; ^*^P < 0.05; ^**^P < 0.01; ^***^P < 0.001 vs. OLETF group.

The serum TC, LDL-C levels, and the liver TC content decreased significantly in OLETF rats upon treatment with FL+MET, but not MET (Figures 2A,C,D). The serum HDL-C level increased in OLETF rats when exposed to FL+MET or MET (Figure 2B), but not significantly. Treatment of OLETF rats with FL+MET, but not MET, significantly increased the fecal TC content (Figure 2E). The liver TG content was significantly decreased in OLETF rats when treated with either FL+MET or MET (Figure 2F).

### Combination of FL and MET influenced the hepatic protein expression

To investigate the molecular mechanisms underlying the preventive action of MET and FL+MET against fat deposition, we measured the phosphorylation or expression levels of hepatic AMPK, SREBP-1c, ACC and HMGCoA reductase, the key proteins involved in lipid metabolism and homeostasis (Figure 2G). Our results showed that the phosphorylation of both AMPK and ACC increased substantially and expression of SREBP-1c moderately decreased in the liver of MET-treated OLETF rats upon co-exposure to FL. Treatment of OLETF rats with both FL and FL +MET reduced the expression of hepatic HMGCoA reductase.

### Combination of FL and MET prevented insulin resistance and improved glucose tolerance

**Figure 3:**
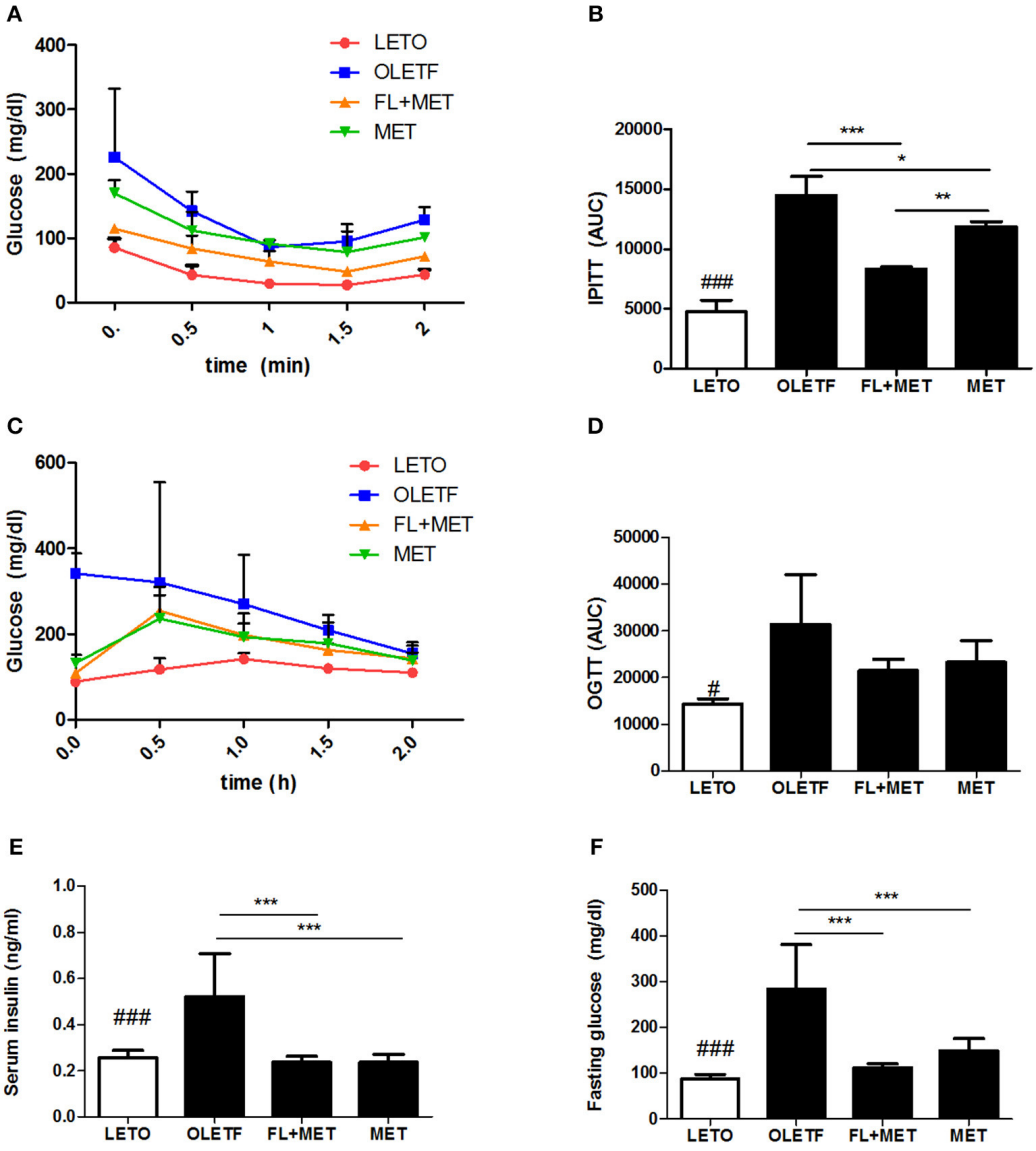
Effect of FL on the insulin sensitivity and glucose homeostasis in OLETF rats. The level of fasting serum glucose **(A)** and area under the curve (AUC) of intraperitoneal insulin tolerance test (IPITT) **(B)** were measured after treatment of the rats with insulin. The level of fasting serum glucose **(C)** and AUC of oral glucose tolerance test (OGTT) **(D)** were determined after the animals were administered glucose. The concentrations of serum insulin **(E)** and glucose **(F)** in the rats sampled at the termination of the experimental duration are depicted. Data are expressed as mean ± SD (*n* = 3). ^#^*P* < 0.05; ^###^*P* < 0.001 vs. LETO group; ^*^P < 0.05; ^**^P < 0.01; ^***^P < 0.001 vs. OLETF group.

We performed the intraperitoneal insulin tolerance test IPITT and OGTT oral glucose tolerance test in the OLETF rats to investigate the effects of MET or FL+MET. The results revealed a time-dependent decrement in fasting serum glucose level up to 1 h post-insulin treatment which in the OLETF rats was most pronounced in FL+MET group followed by MET group (Figure 3A). The area under curve (AUC) of IPITT glucose in OLETF rats decreased significantly in response to treatment with both MET and FL+MET, while AUC of FL+MET group was significantly lower than the MET group (Figure 3B). Our results on OGTT demonstrated a time-dependent decline in fasting serum glucose concentration in all experimental rats throughout the entire 2 h post-glucose administration period, more effectively in both the MET and FL+MET groups (Figure 3C). The AUC of OGTT glucose in OLETF rats insignificantly, but markedly, decreased in response to both MET and FL+MET (Figure 3D). At the time of sacrifice, the serum levels of both fasting insulin and glucose in the MET or FL+MET groups were significantly lower than the OLETF group (Figures 3E,F).

### Combination of FL and MET increased the glucose uptake and insulin secretion in *in vitro* test

**Figure 4:**
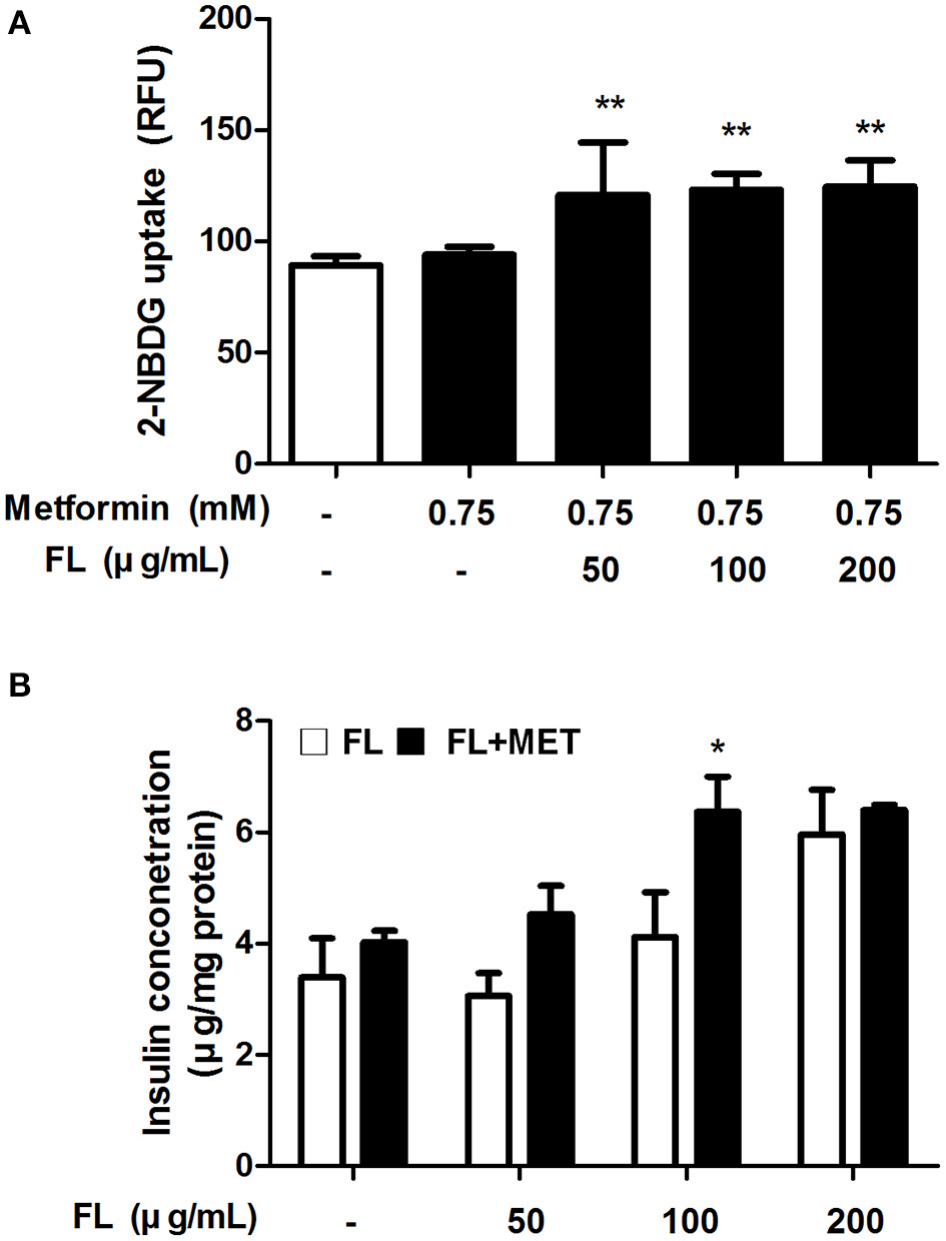
Effect of FL on glucose uptake and insulin secretion in *in vitro* test. Effect of treatment with 0.75 mM MET either alone or in combination with FL at 50, 100, 200 μ g/ml concentrations on the glucose uptake in C2C12 cells **(A)**. Effect of treatment with FL at 50, 100, and 200 μ g/ml concentrations in absence or presence of 0.75 mM MET on the insulin secretion in INS-1 cells **(B)**. Data are expressed as mean ± SD (*n* = 7). ^*^P < 0.05; ^**^P < 0.01 vs. control group.

To verify the beneficial impact FL and MET in combination on the glucose homeostasis, we performed *in vitro* studies using C2C12 myotube and INS-1 cell lines as models. Our results showed that the glucose uptake by C2Cl2 cells treated with 0.75 mM metformin was enhanced significantly upon co-treatment with CL at 50–200 μ g/ml concentrations (Figure 4A). Insulin secretion by INS-1 cells exposed to 50, 100, or 200 μ g/ml of CL increased upon co-treatment with 0.75 mM metformin, significantly at 100 μ g/ml of CL (Figure 4B).

### Combination of FL and MET depleted the marker for liver damage and hepatic oxidative stress

**Figure 5:**
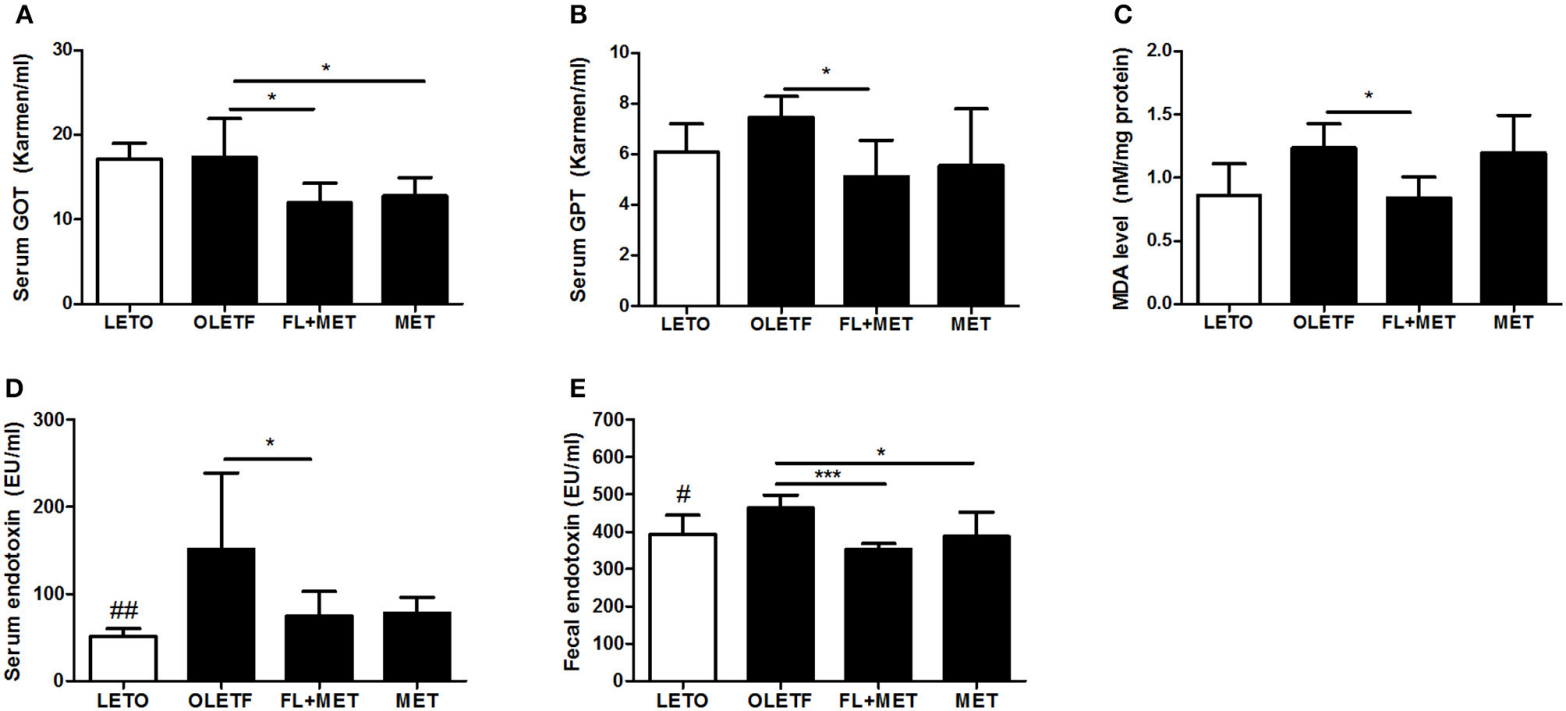
Effect of FL on the liver injury and serum and fecal endotoxin levels in OLETF rats. Serum enzymatic activities of GOT **(A)** and GPT **(B)**, liver lipid peroxidation (MDA level) **(C)**, and serum and fecal endotoxin levels (**D, E**, respectively) in OLETF rats are shown. Data are expressed as mean ± SD (*n* = 7); ^#^*P* < 0.05; ^##^*P* < 0.01 vs. LETO group; ^*^P < 0.05; ^***^P < 0.001 vs. OLETF group.

Treatment of OLETF rats with FL+MET significantly lowered the serum levels of GOT and GPT, the marker enzymes for liver damage and disease, and significantly reduced the hepatic level of MDA, a biomarker for oxidative stress. Exposure of OLETF rats to MET distinctly decreased the serum GOT and GPT level, however only GOT showed statistically significant result (*p* < 0.05), but did not produce any significant effect on the hepatic MDA content (Figures 5A–C).

### Combination of FL and MET reduced the endotoxin level in both serum and feces

Treatment of OLETF rat with FL+MET significantly decreased both the serum and fecal endotoxin levels (Figures 5D,E). Although the exposure of OLETF rats to MET obviously decreased both the serum and fecal endotoxin level (Figures 5D,E), only fecal endotoxin showed statistically significant result (*p* < 0.05).

### Combination of FL and MET shifted the structure and composition of gut microbiota

**Figure 6:**
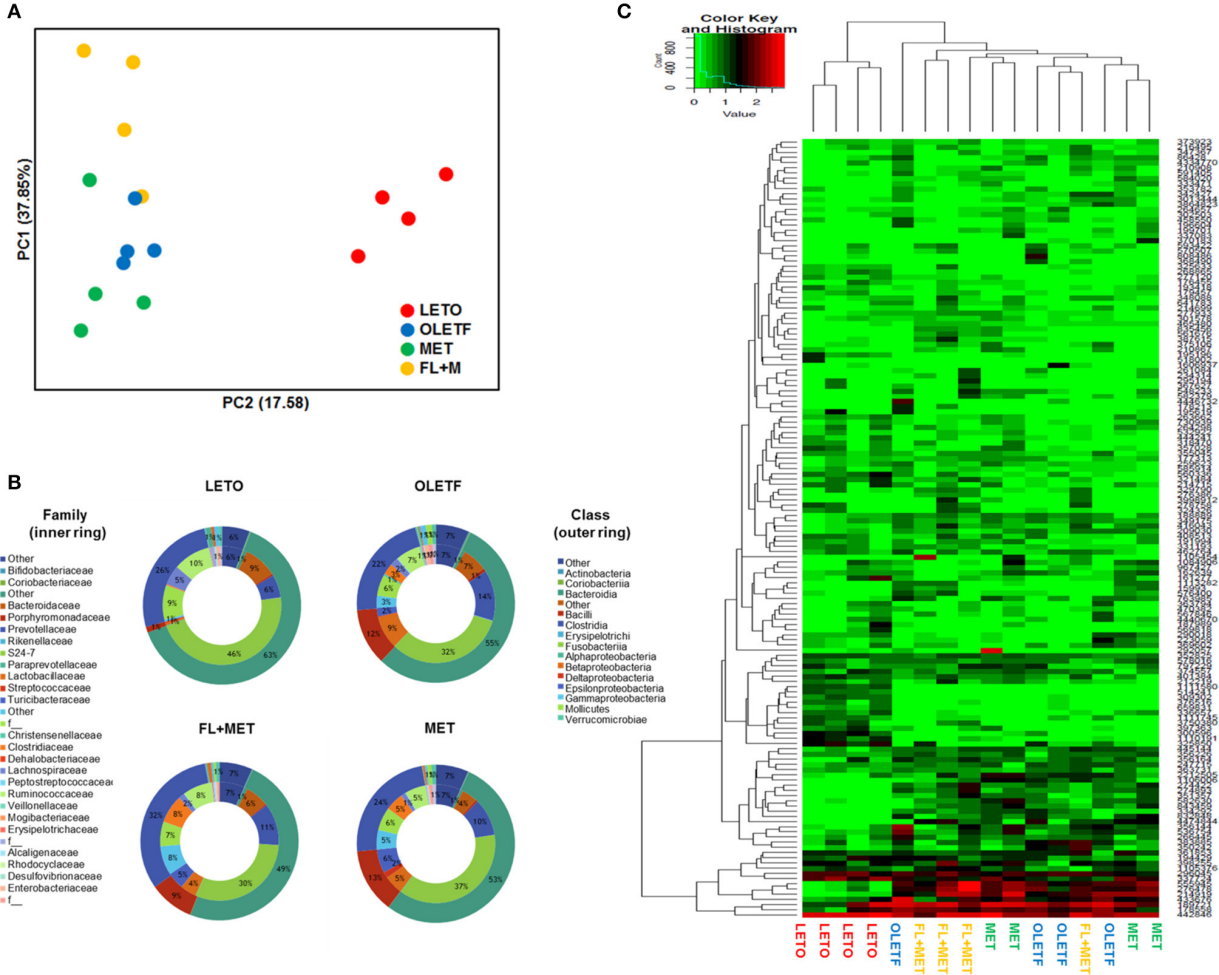
Alteration of structure of gut microbiota by FL in OLETF rats. **(A)** PCoA score plot calculated from OTU levels by QIIME was subjected to unweighted UniFrac analysis. **(B)** Composition profiles of gut microbiota at family and class levels. **(C)** Heatmap and clustering of individual gut microbiota in 150 OTUs.

To profile the effect of FL and FL+MET on gut microbial structure and composition of OLETF rats, 16S rRNA gene sequencing of the stool samples was performed. Our data revealed that the rarefaction per sample was minimal in the LETO group (Figure S2). According to the PCoA of species level composition, the LETO group was clearly separated from the OLETF, FL+MET, and MET groups (Figure 6A). Furthermore, the distance between the OLETF and FL+MET groups was greater than that between the OLETF and MET groups. The microbial composition for each group was further analyzed at the class level. The dominant classes were *Bacteroidia*, contributing to 63.5, 54.8, 48.9, and 53.3%; followed by *Clostridia*, contributing to 25.8, 22.1, 31.7, and 23.7% of the total fecal microbial population of LETO, OLETF, FL+MET, and MET groups, respectively (Figure 6B). Like classes, the microbial composition varied greatly among families. The dominant family was S24-7 in *Bacteroidales* contributing to 46.1, 32.1, 29.9, and 37.1%, followed by *Prevotellaceae* contributing to 6.2, 13.6, 11.2, and 10.14% of the total fecal microbial population of LETO, OLETF, FL+MET, and MET groups, respectively (Figure 6B). Heatmap analysis of 150 OTUs showed that FL+MET group was more closely related to LETO group than MET group (Figure 6C).

**Figure 7:**
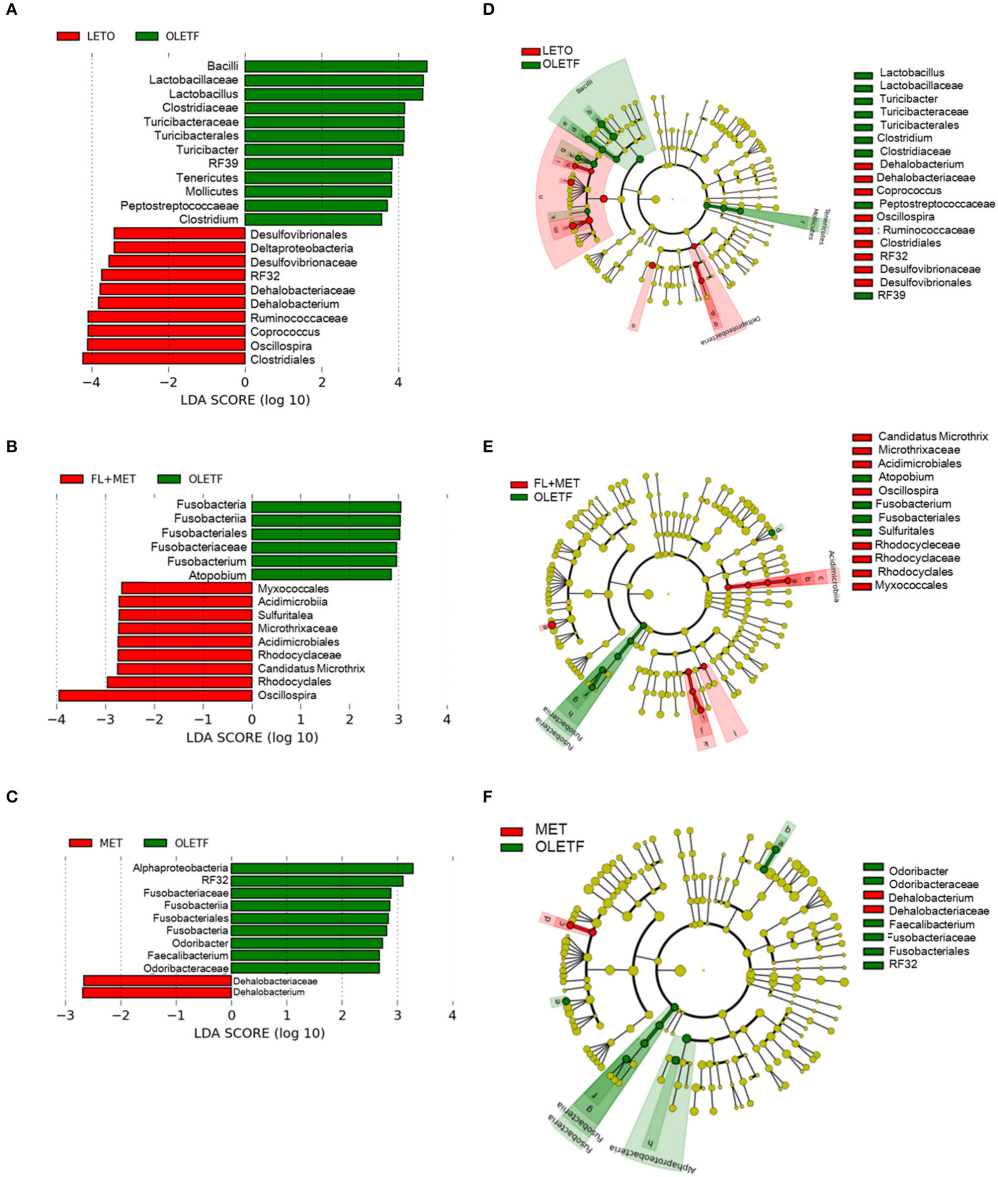
Inter-group variation in the relative abundances of gut microbial communities. Taxonomic comparison of the gut microbiota is demonstrated as follows: LETO vs. OLETF **(A)**, FL+MET vs. OLETF **(B)**, and MET vs. OLETF **(C)**. Circular cladograms depicting the LEfSe results are as follows: LETO vs. OLETF **(D)**, FL+MET vs. OLETF **(E)**, and MET vs. OLETF **(F)**. The alpha value for the factorial Kruskal-Wallis test is <0.05 and the threshold on the logarithmic LDA score for discriminative feature is >2.0.

Next, linear discriminant analysis (LDA) effect size (LEfSe) package was used to determine the differentially relative abundant microbial taxa between the animal groups. The cladogram from the LEfSe results revealed that compared with the OLETF group, 10, 9, and 2 taxa were increased while 12, 6, and 9 taxa were decreased in LETO, FL+MET, and MET groups, respectively (Figure 7). Further, compared with OLETF group, 29, 16, and 8 OTUs were increased while 24, 4, and 7 OTUs were decreased in LETO, FL+MET, and MET groups, respectively (Figure S4). Collectively, these results indicate that the gut microbial composition was differentially modulated in OLETF rats in response to the treatment with MET and FL+MET.

### Disease biomarkers and gut microbiota

**Figure 8:**
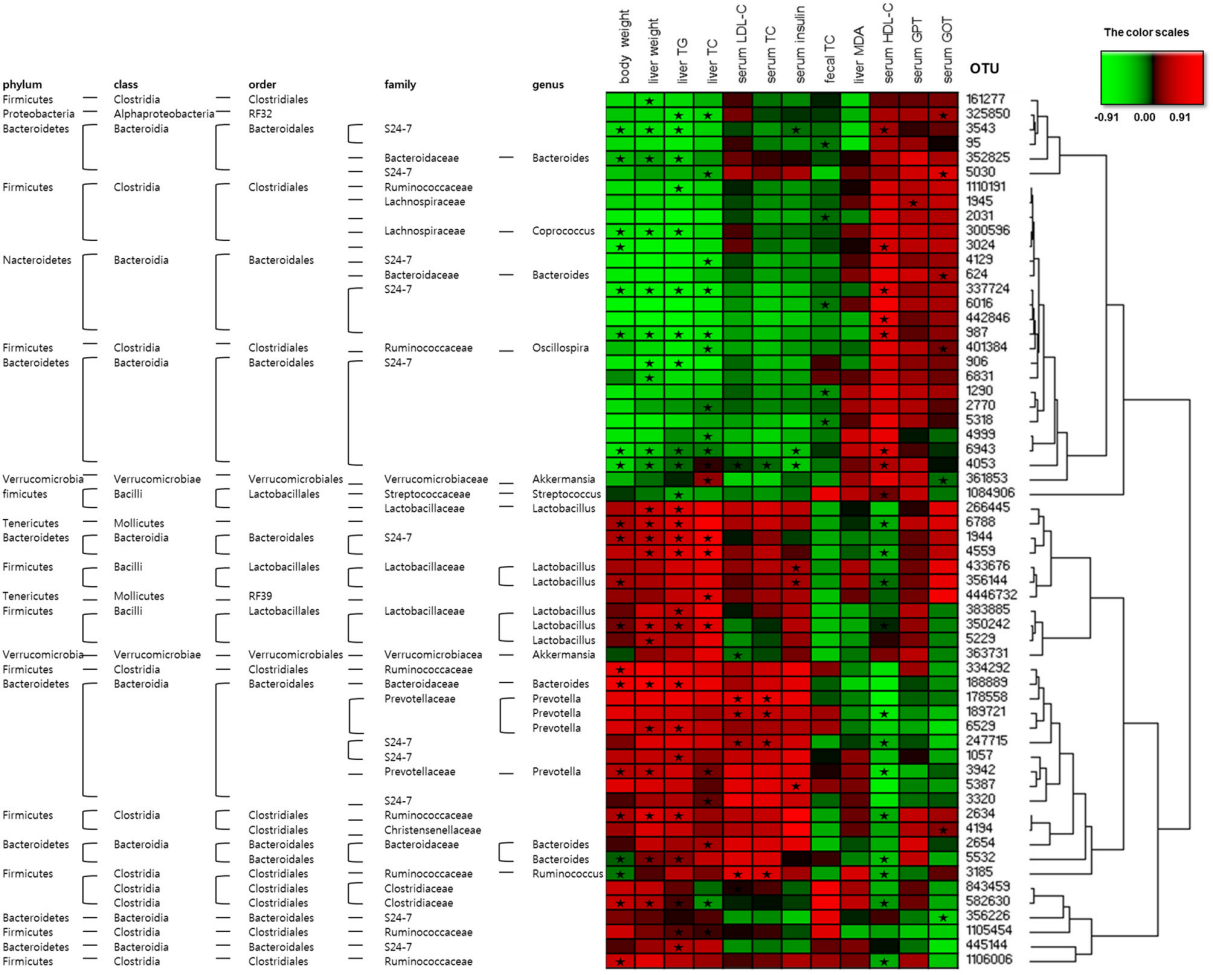
Correlation between gut microbiota and clinical parameters in OLETF rats. Heatmap of correlation between the alterations in gut microbial population and the changes in host parameters related to obesity and metabolic disorders, liver integrity, and lipid peroxidation. Pearson correlation values were used for the matrix. “
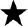
” Denotes adjusted *P* < 0.05.

The heatmap analyses assessed the association between the disease biomarkers and gut microbiota (Figure 8). OTU 337724, OTU 987, OTU 6943, and OTU 4053 were significantly negatively associated and OTU 1944, and OTU 350242 were significantly positively associated with body weight, liver weight, liver TG, and liver TC. OTU 4053 was strongly associated with the biomarkers (8 out of 12), demonstrating significant negative correlation with the body and liver weights, hepatic TG, serum LDL-C, serum TC, and serum insulin levels as well as significant positive correlation with hepatic TC and serum HDL-C levels. OTU 3543, OTU 6943, and OTU 4053 were significantly negatively correlated and OTU 433676, OTU 356144, and OTU 5387 were significantly positively correlated with the serum insulin level (Figure 8).

### Prediction of potential metabolic functions of gut microbiota

**Figure 9:**
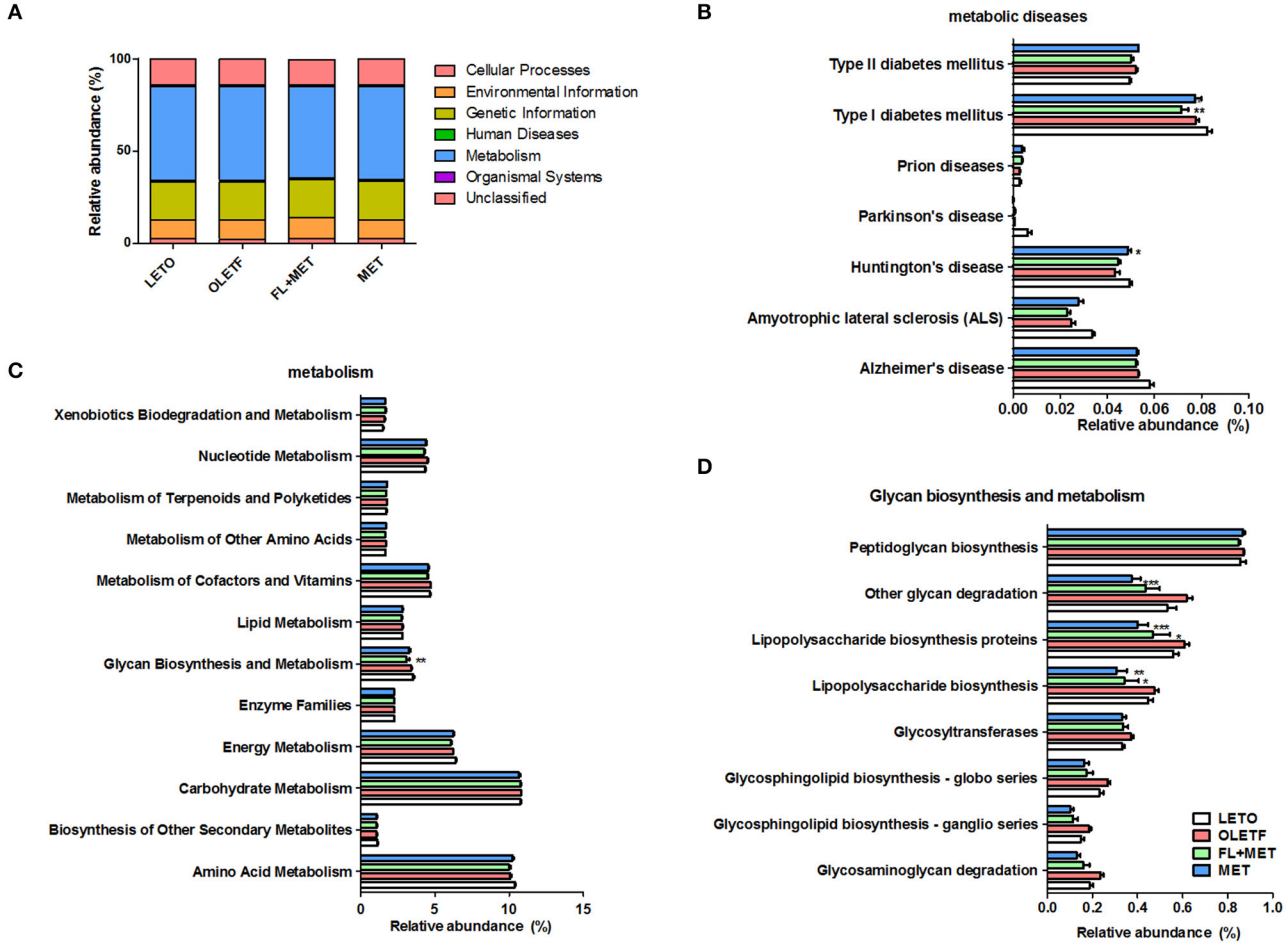
Prediction of metabolic function in OLETF rats. Microbial gene functions in the rats of different experimental groups as indicated using PiCRUSt bioinformatics software package. Results are showing relative abundance of biological entities and characteristics **(A)**, metabolic diseases **(B)**, metabolism **(C)**, and glycan biosynthesis and metabolism **(D)**. Data are expressed as mean ± SED (*n* = 4); ^*^P < 0.05; ^**^P < 0.01; ^***^P < 0.001 vs. OLETF group.

We inferred metagenome functional content using PiCRUSt which predicted several metabolic functions of the gut microbiota. There was no significant difference in the enrichment of metabolic pathways among the experimental animal groups (Figure 9A). However, our results showed that the relative abundance of genes associated with the transporter functional categories including ABC transporters increased more than 2% in OLETF rats in response to FL+MET treatment (Figure S5A). The relative abundance of type I diabetes mellitus associated genes among the bacterial genes related to human metabolic and neurodegenerative diseases were significantly lower in FL+MET group vs. OLETF group (Figure 9B). In metabolic function, most genes associated with glycan biosynthesis and metabolism category were reduced maximally in FL+MET group (Figures 9C,D). Further analysis of this category revealed that many other genes, such as, those associated with glycan degradation, lipopolysaccharide (LPS) biosynthesis, and proteins involved in LPS biosynthesis were significantly reduced in OLETF rats upon treatment with either MET or FL+MET (Figure 9D). Moreover, many genes related to streptomycin biosynthesis and the genes associated with the oxidative phosphorylation category in energy metabolism function were significantly lower in both MET and FL+MET groups compared with OLETF group (Figures S5B,C).

## Discussion

The OLETF rat is a model that is widely used to study NAFLD (Rector et al., 2010), a progressive liver disease characterized by elevated hepatic TG storage and hepatocyte ballooning. In agreement with this, we found an aberrant histological structure of the liver with substantial deposition of fat in the OLETF rats. This is further supported by the biochemical studies revealing higher level of hepatic TC and TG in OLETF rats compared to LETO group.

The histological and biochemical liver profiles revealed that treatment of OLETF rats with FL and MET in combination, but not MET alone, improved hepatosteatosis. Previous studies showed the protective effects of FL or metformin against hepatic steatosis in methionine-choline-deficient diet (MCDD)-fed NASH and diet-induced obese mice models as well as insulin-resistant ob/ob mice with fatty livers (Lin et al., 2000; Woo et al., 2014; Tzeng et al., 2015). Our findings also demonstrated that treatment with FL+MET combination, but not MET alone, reduced both the absolute and relative liver weights of OLETF rats. We propose that FL and MET may act synergistically to reduce liver weight and hepatosteatosis in OLETF rats.

Although cholesterol is important in the physiological processes as a precursor of steroid hormones and bile acids and is an essential component of cell membranes (Morgan et al., 2016), accumulating evidence indicate that hypercholesterolemia could trigger onset and development of NAFLD (Qian et al., 2016). In our study, exposure of OLETF rats to FL+MET combination, but not MET alone, significantly depleted both the hepatic and serum TC contents, serum LDL-C level, and significantly increased the fecal TC content. This is in agreement with our earlier findings on FL-mediated significant lowering of serum lipid parameters in HFD-fed rats treated with LPS (Wang et al., 2014). Chlorogenic acid, one of the major compounds in FL, reduced the serum cholesterol and liver lipid in high-cholesterol diet-fed rats (Wan et al., 2013). Our findings are in alignment with a decline in hepatic expression of HMGCoA reductase, the rate limiting enzyme in cholesterol biosynthesis, in OLETF rats in response to FL+MET treatment.

In the insulin and glucose tolerance tests, as expected, insulin sensitivity and glucose homeostasis were markedly improved in OLETF rats following treatment with metformin. However, the IPITT analysis data demonstrated that decline in AUC (glucose) in MET-treated OLETF animals was potentiated when co-exposed to FL. Additionally, at the time of sacrifice, the serum concentrations of both fasting insulin and glucose in MET or FL+MET groups were significantly lower compared to those of OLETF group. These results agree with our present findings on the beneficial impact of MET and FL combination on the glucose uptake by C2C12 myotubes, a widely used cell culture model for studying insulin action (Xue et al., 2011), as well as on the insulin secretion by INS-1 cells, a suitable model for studying pancreatic beta-cell function (Asfari et al., 1992). It is conceivable that FL+MET combination led to greater improvements compared with MET alone in regulating glucose homeostasis.

AMPK, the function of which has been extensively studied in muscles and liver, is central in maintaining cellular energy homeostasis (Daval et al., 2006; Hardie et al., 2012; Jeon, 2016). Metformin activates AMPK in hepatocytes (Zhou et al., 2001). AMPK induces pathways that augment energy production [glucose transport, fatty acid (FA) oxidation] and switches off pathways that utilize energy (lipogenesis, protein synthesis, gluconeogenesis; Daval et al., 2006). More specifically, AMPK suppresses *de novo* synthesis of FAs, cholesterol and triglycerides, and activates FA uptake and β-oxidation (Jeon, 2016). Activation of AMPK suppresses the gene and protein expression of SREBP-1c (Zhou et al., 2001; Um et al., 2004; You et al., 2004), the key transcription factor regulating fatty acid synthesis. AMPK activation by metformin markedly reduces hepatic steatosis by suppressing SREBP-1c expression (Zhou et al., 2001). Activated AMPK phosphorylates and inactivates ACC, a rate-limiting enzyme of lipogenesis (Hardie et al., 1997), resulting in an induction in FA oxidation and an inhibition in the expression of lipogenic enzymes (Zhou et al., 2001). In our study, the activation of AMPK in terms of its phosphorylation was markedly potentiated in MET-treated OLETF rats by FL. Co-exposure of MET-treated animals to FL moderately reduced the protein expression of SREBP-1c and substantially augmented the phosphorylation of ACC. We surmise that, compared to MET alone, FL+MET combination improved lipid metabolism more in OLETF rats.

Oxidative stress is crucial in the pathogenesis of diabetic complications in OLETF rats (Minamiyama et al., 2007). Our data on MDA measurement revealed an increased hepatic oxidative stress in terms of an enhanced lipid peroxidation in the liver of OLETF rats, consistent with previous observation (Tsuzuki et al., 2015). Our results also demonstrated that an exposure of OLETF rats to FL+MET combination, but not MET, significantly reduced the hepatic content of MDA, agreeing with an antioxidant effect of FL against lipid peroxidation in the liver of dimethylnitrosamine-induced rats (Teng et al., 2010). Both MET and MET+FL significantly reduced the serum GOT, while MET+FL, but not MET alone, significantly lowered the serum GPT in OLETF rats. This is in agreement with findings demonstrating a potent protective effect of FL against dimethylnitrosamine-induced acute liver injury in rats (Teng et al., 2010). In sum, MET+FL combination is more effective than MET alone in protecting the liver against oxidative damage and sustaining the hepatic functions in OLETF rats.

Growing evidences indicate an association of gut microbiota with NAFLD (Zhu et al., 2013; Fukui, 2015; Van Best et al., 2015), a multifactorial condition encompassing genetic, metabolic inflammatory, and environmental factors (Abu-Shanab and Quigley, 2010). Although diet is a vital modulator of the gut microbiota, host genetic factors are important contributors to the shaping of normal gut microbial population (Lynch et al., 2013; Lu et al., 2014). Our PCoA results demonstrated that despite access to similar food, the microbial community in the LETO group varied considerably from that in OLETF groups, supporting the possible involvement of host genetic factors in determining the gut microbial distribution. The samples from OLETF, MET, and MET+FL groups were mainly scattered into three different clusters, with the distance between OLETF and MET+FL groups far more than that between OLETF and MET groups. This suggests that an exposure of OLETF rats to MET causes a modulation in the distribution of gut microbial population, enhanced by co-treatment of the animals with FL. Our findings are in agreement with a recent human metagenomics-based study, revealing significant microbiome alterations in T2D patients in response to metformin treatment (Forslund et al., 2015). Our results are also corroborated by our previous study demonstrating an exposure of HFD-fed or HFD-fed plus LPS-treated rats with FL resulted in a significant alteration in the composition of intestinal flora (Wang et al., 2014).

Emerging repots on characterization and profiling of gut microbiota revealed higher relative abundance of phyla *Bacteroidetes, Proteobacteria*, and *Actinobacteria*, and lower population of phylum *Firmicutes* in subjects suffering from NASH or NAFLD compared to control volunteers (Raman et al., 2013; Wong et al., 2013; Zhu et al., 2013; Michail et al., 2015). In our study, the relative abundance of *Bacteroidetes, Proteobacteria*, and *Actinobacteria* decreased and the population of *Firmicutes* increased in OLETF group upon treatment with MET or FL+MET (Figure S3A–D). Accumulating clinical investigations revealed greater gut microbial relative abundance of the genus *Prevotella, Lactobacillus, Dorea, Blautia*, and *Parabacteroides* and lesser population of genus *Oscillospira, Ruminococcus*, and *Roseburia* in NASH or NAFLD patients compared to control subjects (Raman et al., 2013; Wong et al., 2013; Zhu et al., 2013; Michail et al., 2015; Manco et al., 2017). In the present study, the population of *Prevotella, Lactobacillus, Dorea, Blautia*, and *Parabacteroides*, and OTU 363731 decreased and the relative abundance of *Oscillospira, Ruminococcus*, and *Roseburia* increased in OLETF rats upon exposure to FL+MET. Except for *Parabacteroides* and *Ruminococcus*, gut microbiota of OLETF group responded similarly when treated with only MET (Figure S3). In parallel, our analysis revealed that *Prevotella* and *Lactobacillus* were significantly associated with the typical symptoms of NAFLD. OTU 363731, identified in our study as *Akkermansia muciniphila*, has been reported as T2D-enriched bacteria (Figure S3M; Qin et al., 2012; Shin et al., 2013). Taking our findings and previous literature (Abu-Shanab and Quigley, 2010) together, it is conceivable that MET or FL counteract the changes in gut microbial community associated with NASH or NAFLD.

We performed taxonomic and functional annotation of gut microbial communities and determined the tentative metagenomes from phylogenetically associated reference genomes. Based on evolutionary modeling and using a reference genome database, PiCRUSt was employed to infer metagenomes functional content from 16S rRNA gene function data (Langille et al., 2013). The relative abundance of genes related to LPS biosynthetic pathway and the proteins involved in LPS biosynthesis were higher in OLETF vs. LETO groups. However, the relative abundance of these microbial genera were significantly lower in both the MET and MET+FL groups compared to OLETF rats. This phenomenon was further confirmed by our experimental studies demonstrating reduction in serum and fecal endotoxin content in OLETF rats in response to the treatments with MET or MET+FL, more pronouncedly by the latter treatment. Growing evidence indicate that metabolic endotoxins like LPS initiates obesity and insulin resistance (Cani et al., 2007) and is important in the pathogenesis of NASH and NAFLD (Zhu et al., 2015).

Our study demonstrated a differential relative abundance of genes among the experimental groups that are related to biosynthetic pathways of secondary metabolites including antibiotics like streptomycin, penicillin, and novobiocin. This might influence the differential distribution pattern of the gut microbial population among the study groups through the inhibition of growth of selected bacterial community.

The present study proposes that a combination of MET and FL have the potential to combat T2D as well as NAFLD. More specifically, FL+MET treatment produces synergetic effects to attenuate serum and liver cholesterol as well as liver oxidation and hepatic damages in an animal model of NAFLD. To the best of our knowledge, this is the first report that provides the gut microbial composition of OLETF rats. The present study assesses significant correlations among the metabolic markers and bacterial OTUs, as supported by PiCRUSt that predicts several metabolic functions of the gut microbiota from marker genes such as, 16S rRNA genes. In sum, it is conceivable that combined treatment of FL and MET could improve glycometabolism and hepatic lipometabolism as well as liver injury induced by metabolic diseases such as, obesity and T2D.

## Author contributions

YC, HC, and HK conceived the study, contributed to the experimental designs, and interpreted the results. NS, J-HW, AA, and S-KL performed the experiments, and analyzed the results. NS and J-HW generated the figures. SB conceived the study and interpreted the results. NS and SB wrote the paper. All authors read, discussed the results and approved the final manuscript.

### Conflict of interest statement

The authors declare that the research was conducted in the absence of any commercial or financial relationships that could be construed as a potential conflict of interest.

## Supplementary material

Figure S1 HPLC fingerprint of FL. Chromatograms of standard **(A)** and FL extract **(B)** are shown. 1. chlorogenic acid; 2. caffeic acid.

Figure S2 Diversity richness of the gut microbial communities in the rats of different experimental groups as indicated. Rarefaction measurement of each sample **(A)** and average **(B)**.

Figure S3 Relative abundance of gut microbial OTUs at phylum level [*Bacteroidetes* **(A)**, *Firmicutes* **(B)**, *Proteobacteria* **(C)**, and *Acinobacteria* **(D)**], genus level [*Prevotella* **(E)**, *Lactoabacillus* **(F)**, *Dorea* **(G)**, *Parabacteroides* **(H)**, *Blautia* **(I)***, Oscillospira* **(J)**, *Ruminococcus* **(K)**, *Roseburia* **(L)**], and for OTU353731 **(M)**. Data are expressed as mean ± SED (*n* = 4).

Figure S4 Inter-group variation in the relative abundance of gut microbial communities. Taxonomic comparison of the gut micobiota as follows: LETO vs. OLETF **(A)**, FL+MET vs. OLETF **(B)**, and MET vs. OLETF **(C)**. The alpha value for the factorial Kruskal-Wallis test is <0.05 and the threshold on the logarithmic LDA score for discriminative feature is >2.0.

Figure S5 Prediction of relative abundance of major metabolism functions in the rats of different experimental groups as indicated using PiCRUSt bioinformatics software package. Data are shown for metabolites with over 2% relative abundance **(A)**, biosynthesis of other secondary metabolites **(B)**, and energy metabolisms **(C)**. Data are expressed as mean ± SED (*n* = 4). ^*^*P* < 0.05; ^**^*P* < 0.01; ^***^*P* < 0.001 vs. OLETF group.
