# Supplementary figures and images for: Flos Lonicera Combined with Metformin Ameliorates Hepatosteatosis and Glucose Intolerance in Association with Gut Microbiota Modulation
Source: Front Microbiol. 2017 Nov 17;8:2271. doi: 10.3389/fmicb.2017.02271 (PMC5698303; doi:10.3389/fmicb.2017.02271)

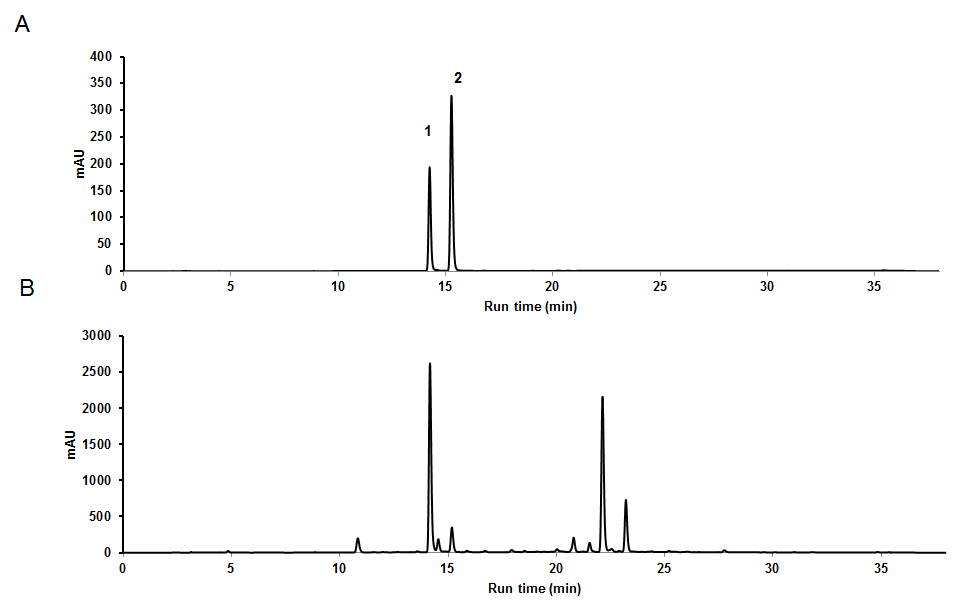

Supplement: Figure S1 — HPLC fingerprint of FL. Chromatograms of standard (A) and FL extract (B) are shown. 1. chlorogenic acid; 2. caffeic acid. [file Image1.TIF]

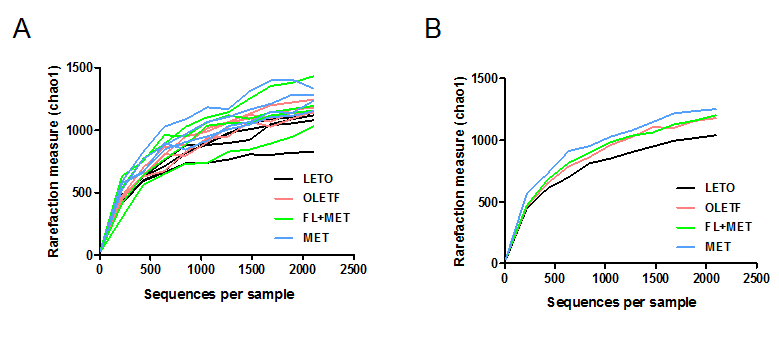

Supplement: Figure S2 — Diversity richness of the gut microbial communities in the rats of different experimental groups as indicated. Rarefaction measurement of each sample (A) and average (B). [file Image2.TIF]

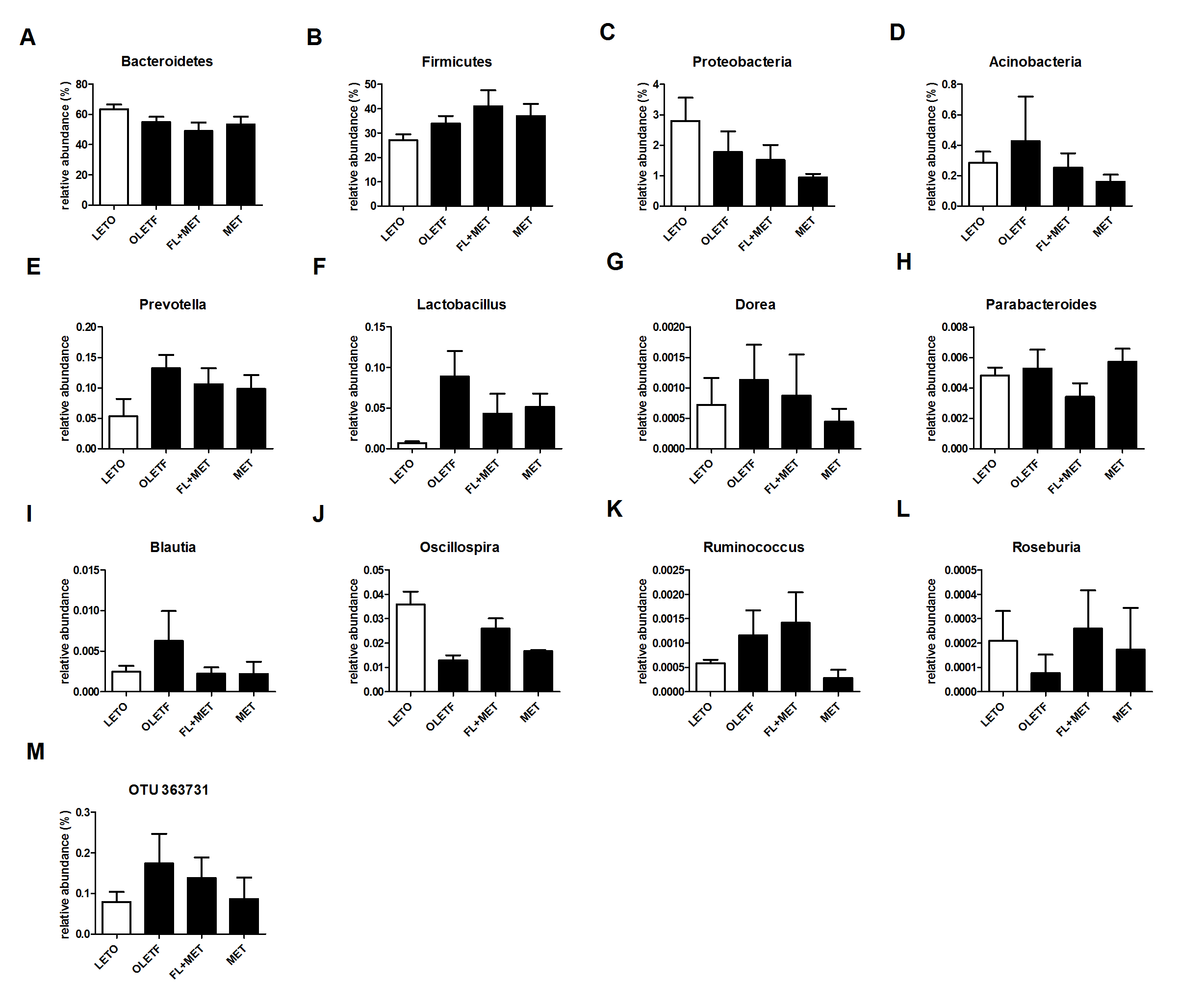

Supplement: Figure S3 — Relative abundance of gut microbial OTUs at phylum level [Bacteroidetes (A), Firmicutes (B), Proteobacteria (C), and Acinobacteria (D)], genus level [Prevotella (E), Lactoabacillus (F), Dorea (G), Parabacteroides (H), Blautia (I), Oscillospira (J), Ruminococcus (K), Roseburia (L)], and for OTU353731 (M). Data are expressed as mean ± SED (n = 4). [file Image3.TIF]

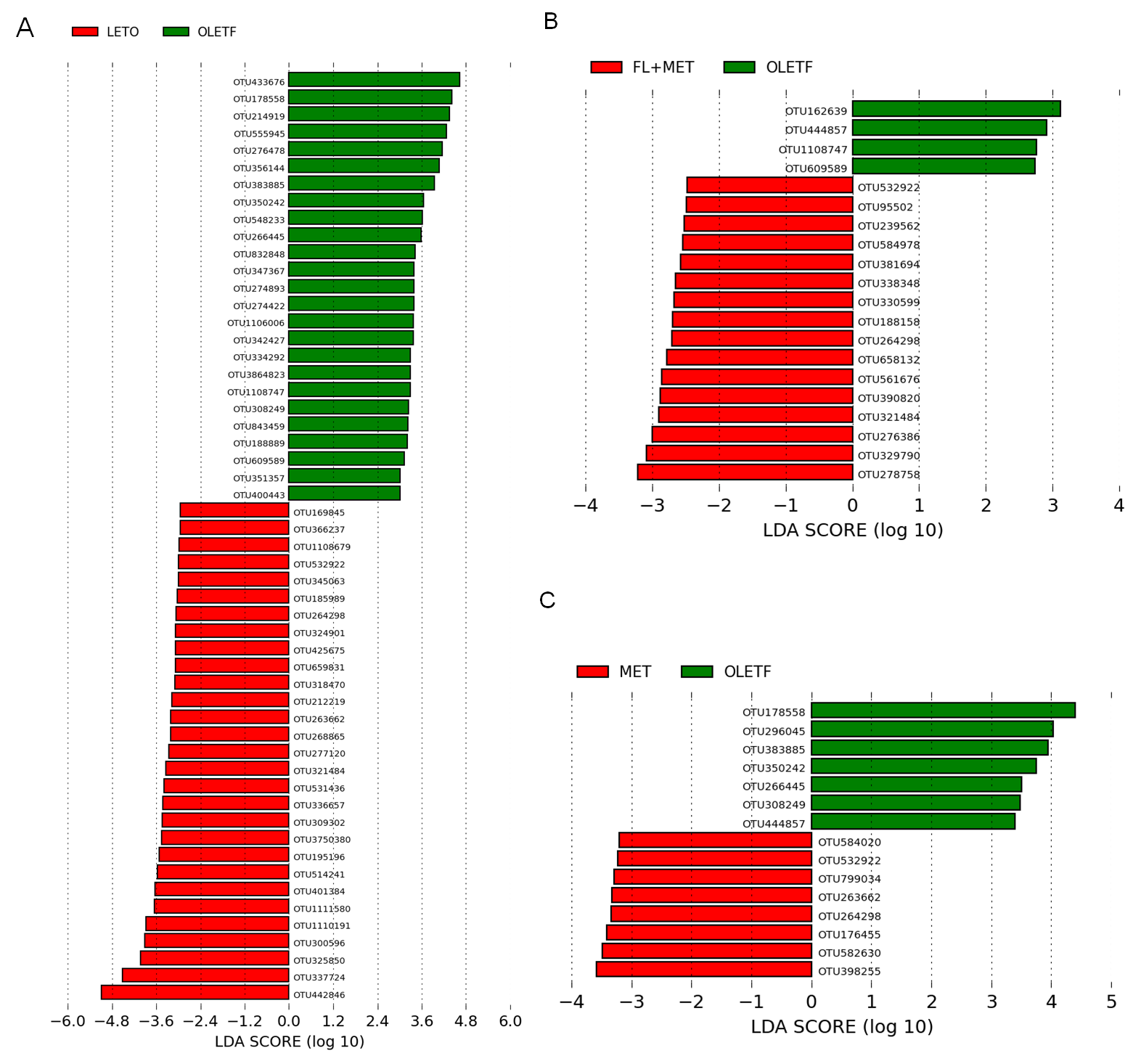

Supplement: Figure S4 — Inter-group variation in the relative abundance of gut microbial communities. Taxonomic comparison of the gut micobiota as follows: LETO vs. OLETF (A), FL+MET vs. OLETF (B), and MET vs. OLETF (C). The alpha value for the factorial Kruskal-Wallis test is <0.05 and the threshold on the logarithmic LDA score for discriminative feature is >2.0. [file Image4.TIF]

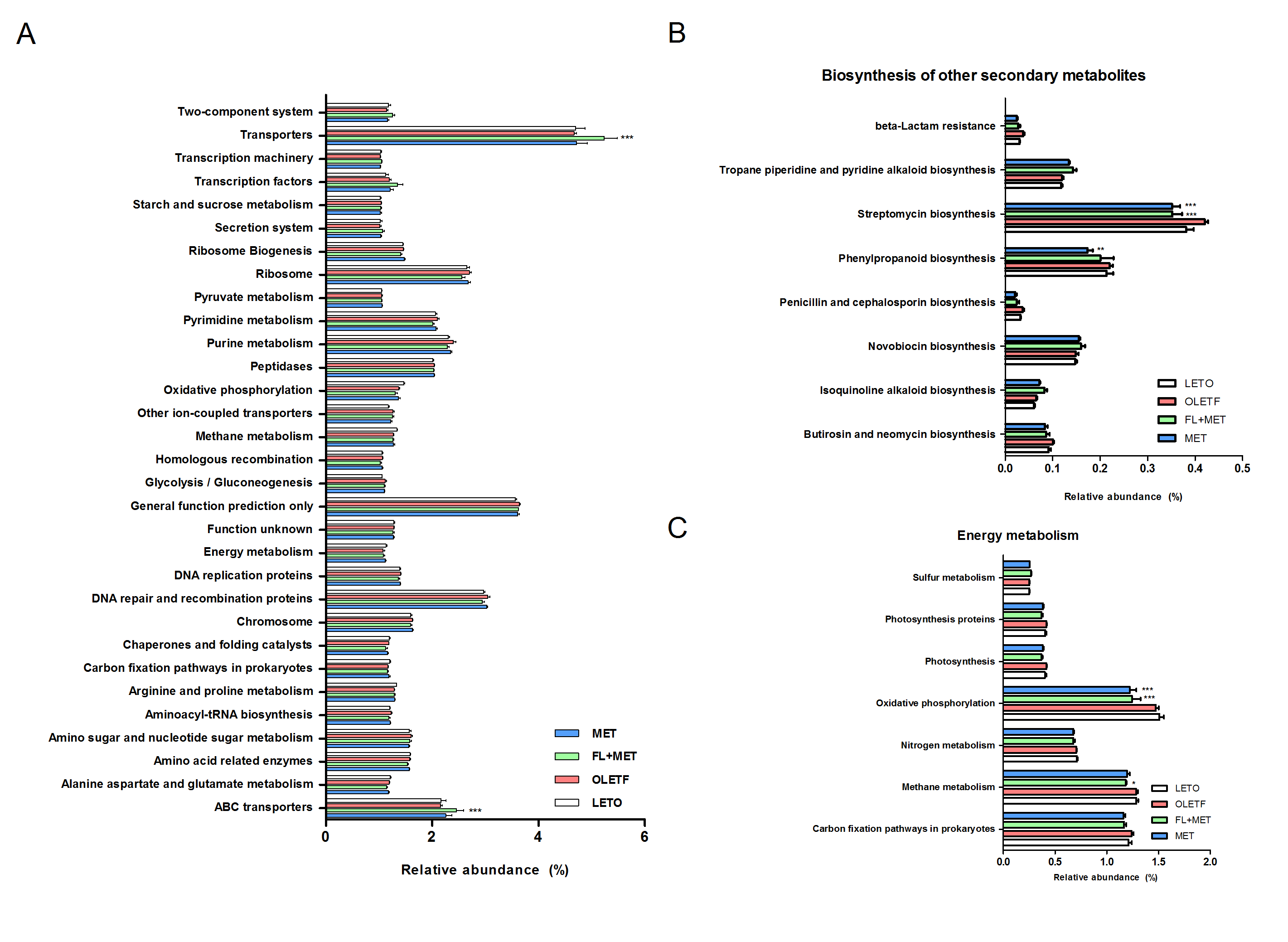

Supplement: Figure S5 — Prediction of relative abundance of major metabolism functions in the rats of different experimental groups as indicated using PiCRUSt bioinformatics software package. Data are shown for metabolites with over 2% relative abundance (A), biosynthesis of other secondary metabolites (B), and energy metabolisms (C). Data are expressed as mean ± SED (n = 4). *P < 0.05; **P < 0.01; ***P < 0.001 vs. OLETF group. [file Image5.TIF]
